# Racial disparities and prevalence of cardiovascular disease risk factors, cardiometabolic risk factors, and cardiovascular health metrics among US adults: NHANES 2011–2018

**DOI:** 10.1038/s41598-022-21878-x

**Published:** 2022-11-14

**Authors:** Stephanie M. Lopez-Neyman, Kathleen Davis, Namvar Zohoori, K. Shane Broughton, Carolyn E. Moore, Derek Miketinas

**Affiliations:** 1 Department of Nutrition Sciences, Texas Woman’s University, Denton, TX USA; 2 Department of Epidemiology, Fay W. Boozman College of Public Health, University of Arkansas for Medical Sciences, Little Rock, AR USA; 3 Department of Nutrition Sciences, Texas Woman’s University, Houston, TX USA

**Keywords:** Biomarkers, Cardiology, Diseases, Risk factors

## Abstract

This study estimated the prevalence of cardiovascular disease (CVD) risk factors, cardiometabolic (CM) risk factors, and cardiovascular health metrics (CVHMs) among US adults and across race/ethnicity groups. The study comprised 8370 US adults aged ≥ 20 years from the National Health and Nutrition Examination Survey (NHANES) 2011–2018, free of coronary heart disease/heart failure, angina/angina pectoris, heart attack, and stroke, who provided complete data for the outcome variables of interest. Age-adjusted prevalence of CVD and CM risk factors, and CVHMs were computed for all adults and across race/ethnicity groups. All analyses accounted for the complex, multi-stage survey sampling design of the NHANES. Hypertension (45.0%), obesity (40.0%), fasting plasma glucose ≥ 100 mg/dL or hypoglycemic medication (51.0%), ideal physical activity (59.2%) and ideal smoking status (56.9%) were most prevalent for the whole sample. Mexican Americans and non-Hispanic Blacks had elevated risk for some, but not all, CVD and CM risk factors compared to non-Hispanic Whites and non-Hispanic Asians. Reducing further health disparities and persisting differences among racial and ethnic groups is vital to achieving the American Heart Association vision of all people having ideal cardiovascular health, living healthier and longer.

## Introduction

The American Heart Association’s (AHA) new mission and 2030 Impact Goal focuses on healthy life expectancy regardless of race and ethnicity, economic status, or other demographic or geographic characteristics^1^. Cardiovascular and cardiometabolic risk factors, behavioral risk factors, environment, and disease prevalence are critical to monitoring progress toward the 2030 Impact Goal^1^. Given that cardiovascular disease (CVD) is more prevalent among the uninsured, the aging, and all major racial and ethnic groups monitoring in these groups is critical^2^.

Population health monitoring can occur through surveillance and continuous analysis of health-related data^3^. Surveys like the National Health and Nutrition Examination Survey (NHANES), registries, and cohort studies are examples of current surveillance efforts for CVD^3^. Data collection from NHANES provides data for examining the prevalence of estimates of CVD and cardiometabolic risk factors^4–8^ and cardiovascular health^9,10^ for the whole US population and major population subgroups. Briefly, cardiometabolic risk factors like increased waist circumference, insulin resistance, or inflammation measured by high-sensitivity C-reactive protein (hsCRP) increase the likelihood of diabetes and/or CVD development^11^. In addition, CVD risk factors such as tobacco use and an unhealthy diet increase the likelihood of CVD development^12^. Cardiovascular health involves concepts in health and promotion, using a construct of ideal cardiovascular health for monitoring cardiovascular health over time^13^. Additionally, ideal cardiovascular health encompasses health behaviors (diet quality, physical activity, weight, and current smoking) and health factors (total cholesterol, blood pressure, and fasting plasma glucose)^13^.

Health organizations and government agencies collaboratively work on releasing reports providing the most up-to-date information related to cardiovascular risk factors for the US population from pooled data sources such as NHANES^12,14^. However, they do not examine both CVD risk factors and cardiovascular health together with cardiometabolic risk factors. It would be beneficial to examine CVD risk factors and cardiovascular health jointly with cardiometabolic risk factors. Those risk factors lead not only to the development of diabetes but also to CVD. Furthermore, examining the prevalence of estimates of CVD and cardiometabolic risk factors and cardiovascular health together provides a better understanding of the health of the whole US population and major population subgroups to achieve the AHA 2030 Impact Goal. Thus, this study aims to comprehensively assess and report the prevalence of CVD and cardiometabolic risk factors and cardiovascular health estimates for a nationally representative sample and across race and ethnic groups using NHANES, 2011–2018.

## Methods

### Study design and study population

NHANES is an ongoing, cross-sectional survey that monitors the health and nutritional status of the US civilian, noninstitutionalized population. The sample selection entails using a complex, stratified, multistage probability design^15^, with written and informed consent obtained from each participant. The National Center for Health Statistics Research Ethics Review Board approved the NHANES study protocol^16^. The researcher’s Institutional Review Board (IRB) determined this secondary data analysis was exempt from review. All methods were performed in accordance with relevant guidelines and regulations and followed the Strengthening the Reporting of Observational Studies for Epidemiology (STROBE) guidelines for cross-sectional studies^17^.

**Figure 1:**
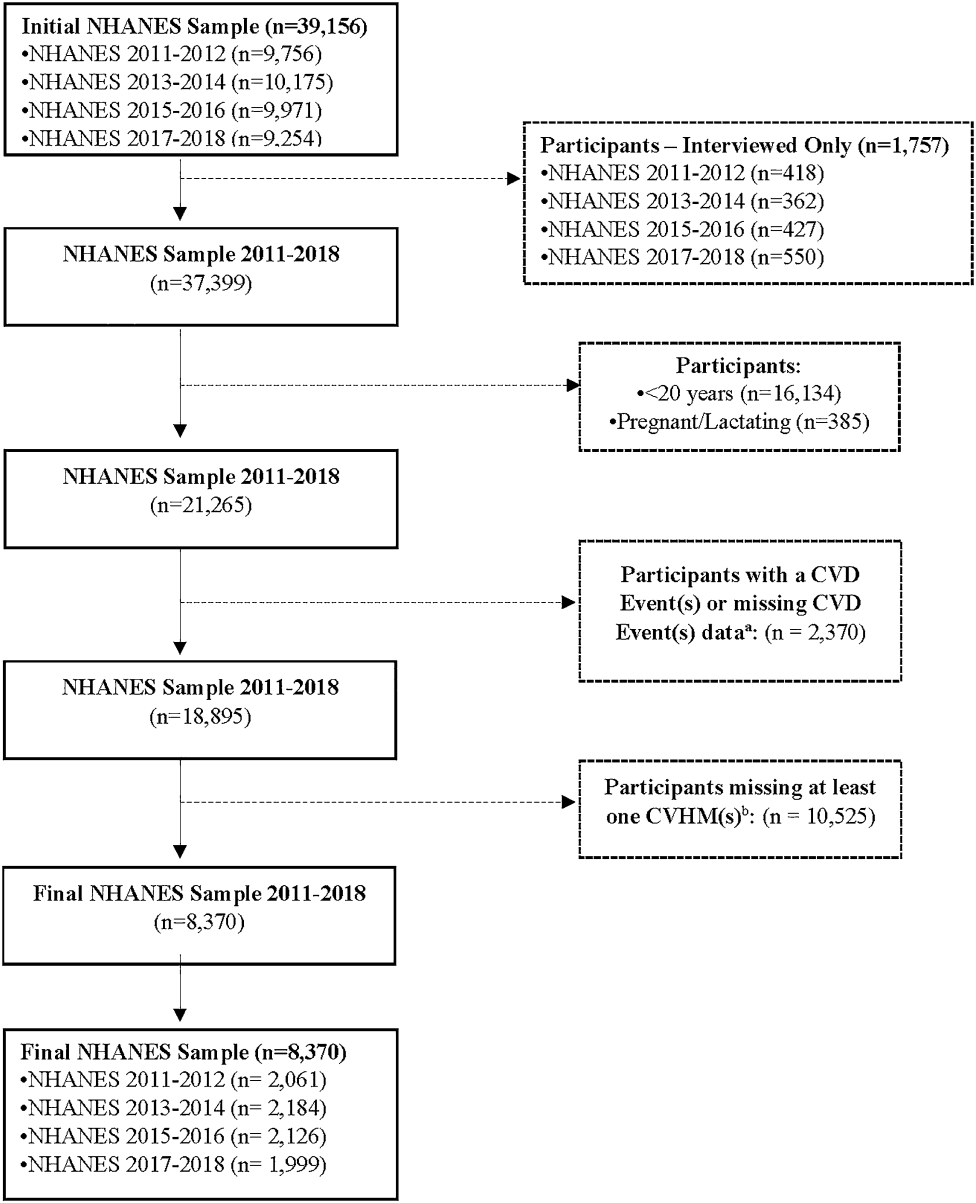
Flow chart of participant selection. *NHANES* National Health and Nutrition Examination Survey, *CVD* cardiovascular disease, *CHF* coronary heart failure, *CHD* coronary heart disease, *ANG* angina/angina pectoris, *HAT* heart attack, *CVHM* cardiovascular health metric, *PA* physical activity, *SS* smoke status, *BMI* body mass index, *BP* blood pressure, *CHOL* total cholesterol, *FPG* fasting plasma glucose. ^a^CVD events includes CHF, CHD, ANG, HAT, Stroke. ^b^CVHM includes PA, SS, BMI, BP, CHOL, FPG.

This analysis used data from the 2011 to 2018 NHANES (n = 39,156 participants). The analysis sample included non-pregnant, non-lactating adults 20 years and older who had measures for CVD and cardiometabolic risk factors and were free of coronary heart disease/heart failure, angina/angina pectoris, heart attack, and stroke (n = 18,895). We excluded participants who had incomplete information from the seven cardiovascular health metrics (CVHMs), yielding a final analysis sample size of 8370 participants (Fig. 1).

### Demographic characteristics

We analyzed demographic information from the NHANES participants’ home interviews, including age, gender, race and ethnicity, education level, health insurance, current smoking status, and the poverty income ratio (PIR). We categorized the demographics as follows: age (20–39 years, 40–59 years, and ≥ 60 years); race and ethnicity (self-reported) non-Hispanic white, non-Hispanic black, Mexican American, other Hispanic, and non-Hispanic Asian; education (< 9th grade, 9th to 12th grade [but no high school diploma (HSD)], HSD or general education degree [GED], some college or associate degree, and ≥ bachelor’s degree); PIR, calculated by dividing the individual or family income by the US poverty guidelines specific to the survey year, and categorized as low income (PIR < 1.3), middle income (PIR 1.3 to < 3.5), and high income (PIR > 3.5)^18^. In addition, we included self-reported medication use for high blood pressure, diabetes, and high cholesterol.

### Measurement of risk factors and cardiovascular health metrics

We used the mean of all provided systolic blood pressure (SBP) and diastolic blood pressure (DBP) measurements using the standard NHANES protocol^19^. Trained personnel in the NHANES mobile examination center followed standards to obtain weight and height and calculated body mass index (BMI), weight in kilograms/height in meters^2^^19^. Smoking status was assessed from NHANES participants’ responses to the two variables, SMQ020 (smoked at least 100 cigarettes in life) and SMQ040 (do you now smoke cigarettes), categorized as current, former, and never smokers^19^. Laboratory values were collected using standard NHANES protocols^19^. We used non-fasted participants’ total cholesterol, high-density lipoprotein cholesterol (HDL-C), high-sensitivity C-reactive protein (hsCRP), fasted participants’ triglycerides, fasting blood glucose, and insulin. Homeostatic Model Assessment-Insulin Resistance (HOMA-IR) was calculated as fasting glucose (mg/dL) × fasting insulin (mU/L/405)^20^.

The Healthy Eating Index-2015 (HEI-2015) measures diet quality based on the 2015–2020 Dietary Guidelines for Americans^21^. The HEI-2015 includes 13 components, nine focusing on nutrient adequacy (e.g., total fruit, whole grains, and dairy) and four focusing on moderation (e.g., sodium and saturated fats). Each component has a designated maximum score of 5 or 10 points, which are summed for a maximum score of 100 points, indicating perfect adherence to the Dietary Guidelines for Americans 2015–2020. NHANES assesses physical activity using participants’ questionnaire responses regarding the frequency and duration of moderate to vigorous work and recreational activities^19^.

### Cardiovascular disease risk factors

We included five CVD risk factors in our analysis: hypertension, hypercholesterolemia, obesity, diabetes, and smoking. We defined the CVD risk factors as the following (dichotomous variables [1 = present, 0 = absent]): hypertension, as mean SBP ≥ 130 mmHg, mean DBP ≥ 80 mmHg, or antihypertensive medication use^22^; hypercholesterolemia, as total cholesterol ≥ 240 mg/dL, or lipid-lowering medication use; obesity, BMI ≥ 30 kg/m^2^; and diabetes, as a fasting plasma glucose as ≥ 126 mg/dL, hemoglobin A1c (HbA1c) ≥ 6.5%, or hypoglycemic medication use. We classified participants as current or non-smokers. Each CVD risk factor was assigned a score of 1, indicating the participant had the CVD risk factor. We then summed the number of CVD risk factors for each participant, ranging from 0 to 5.

### Cardiometabolic risk factors

We included five cardiometabolic risk factors in the analysis: low HDL-C, elevated triglycerides, glucose, HOMA-IR, and hsCRP. We defined the cardiometabolic risk factors as the following (dichotomous variables [1 = present, 0 = absent]): HDL-C < 40 mg/dL (men) and < 50 mg/dL (women) [in the context of metabolic syndrome, low HDL-C cut points differ for men and women; we elected not to use the low HDL-C cut point of < 40 mg/dL for both men and women]^23^; triglycerides ≥ 150 mg/dL; fasting plasma glucose ≥ 100 mg/dL or hypoglycemic medication use; HOMA-IR ≥ 2.5^20^; and hsCRP > 3.0 mg/L^24^. Each cardiometabolic risk factor was assigned a score of 1. A score of 1 indicated the person had a cardiometabolic risk factor. We then summed the number of cardiometabolic risk factors (low HDL-C, elevated triglycerides, and glucose) for each participant, ranging from 0 to 3. We omitted HOMA-IR and hsCRP in the summation of the cardiometabolic risk factors because the data were not available for NHANES survey cycles 2011–2015.

### Cardiovascular health metrics

The AHA defined seven CVHMs, behavior (diet, physical activity, smoking status, BMI) and health factors (blood pressure, total cholesterol, and fasting plasma glucose) (Table 1)^13^. The levels for each metric include poor (a score of 0), intermediate (a score of 1), or ideal (a score of 2). We scored CVHMs for each health behavior and factor (CVHM): 0 points (poor), 1 point (intermediate), or 2 points (ideal)^25^. We then summed each participant’s CVHM, creating a cardiovascular health (CVH) composite score ranging from 0 to 14 points, and then categorized participants into three CVH groups: poor (0–4 points), intermediate (5–9 points), and ideal (10–14 points)^26^.

**Table 1:** Definitions of cardiovascular health metrics.

Metric	Ideal (Score = 2)	Intermediate (Score = 1)	Poor (Score = 0)
**Behaviors**			
Smoking status	Never smoke	Former smoker	Current smoker
BMI	BMI < 25 kg/m^2^	BMI 25–29.9 kg/m^2^	BMI ≥ 30 kg/m^2^
Diet score	HEI > 69.3	HEI 56.9–69.3	HEI < 56.9
Physical activity	≥ 150 min/wk moderate or ≥ 75 min/wk vigorous or ≥ 150 min/wk moderate + vigorous; physical activities included	1–149 min/wk moderate or 1–74 min/wk vigorous or 1–149 min/wk moderate + vigorous; physical activities included	None
**Health factors**			
Total cholesterol	< 200 mg/dL	200–239 mg/dL or treated to goal	≥ 240 mg/dL
Blood pressure	SBP < 120 and DBP < 80 mmHg	SBP 120–129 and DBP < 80 mmHg or treated to goal	SBP ≥ 130 or DBP ≥ 80 mmHg
Fasting plasma glucose	< 100 mg/dL	100–125 mg/dL or treated to goal	≥ 126 mg/dL

*BMI* body mass index, *HEI* healthy eating index, *SBP* systolic blood pressure, *DBP* diastolic blood pressure.

### Statistical analyses

All analyses were adjusted to account for the complex, multistage survey sampling design of the NHANES. We expressed data as mean (95% confidence interval). We calculated the age-adjusted prevalence of CVD and cardiometabolic risk factors and CVHMs for the total sample and each race and ethnic groups. We used the 2010 US Census population estimates for age-adjustment^27^; the proportion of adults aged 20–39 years, 40–59 years and ≥ 60 years was 36.7%, 37.9%, and 25.3%, respectively. We conducted pairwise comparisons of age-adjusted CVD and cardiometabolic risk factors, CVHMs, and CVH groups to determine which race and ethnic groups differed significantly. We used Bonferroni adjustment in the pairwise comparisons to account for multiple testing. Linear models were fit to estimate trends in the proportions or means of CVD and cardiometabolic risk factors and CVH metrics across survey cycles. All statistical tests were 2-sided, with a P-value of < 0.05 statistically significant. We conducted all analyses using SAS software, version 9.4 (SAS Institute, Cary, NC).

## Results

### Participant characteristics

This representative sample of US adults from NHANES consisted of predominately non-Hispanic white adults (66.0%) and had equal representation of men and women (49.6% and 50.4%, respectively). The mean age of the participants was 49.2 years. Mexican Americans (13.8%) and other Hispanics (17.5%) tended to have less representation in the ≥ 60 years age group than non-Hispanic whites (30.5%). Non-Hispanic Asians (57.6%) and non-Hispanic whites (34.9%) obtained the highest levels of education. Mexican Americans (43.1%) and other Hispanics (28.7%) were most likely to have less than high school education. Blood-pressure lowering and diabetes medication use were higher among non-Hispanic blacks (33.1% and 17.9%, respectively), while lipid-lowering medication use was higher among non-Hispanic whites (20.4%) (Table 2).

**Table 2:** Demographic characteristics for adults ≥ 20 years by race/ethnicity.

Characteristics	Total^a^ (n = 8370)	Mexican American (n = 1220)	Other Hispanic (n = 898)	Non-Hispanic White (n = 3097)	Non-Hispanic Black (n = 1822)	Non-Hispanic Asian (n = 1046)
**Gender**						
Men	49.6 (48.3, 50.9)	53.2 (50.4, 55.8)	50.5 (47.1,53.8)	50.2 (48.2, 52.0)	44.1(42.1,47.0)	47.9 (45.3, 50.6)
Women	50.4 (49.1, 51.7)	46.8 (44.1, 49.5)	49.5 (46.1, 52.8)	49.8 (47.9, 51.7)	55.5 (52.9, 57.8)	52.1 (49.3, 54.6)
**Age group**						
20–39 years	34.5 (32.7, 36.4)	49.6 (45.0, 54.1)	43.9 (39.9, 47.8)	29.8 (27.5, 32.1)	38.7 (35.9, 41.5)	43.1 (38.9, 47.4)
40–59 years	39.1 (37.3, 40.9)	36.6 (33.4, 39.6)	38.6 (35.2, 42.0)	39.7 (37.4, 41.8)	40.2 (36.9, 43.4)	34.6 (31.5, 37.7)
≥ 60 years	26.4 (24.6, 28.1)	13.8 (10.8, 16.8)	17.5 (14.5, 20.4)	30.5 (28.2, 32.7)	21.0 (19.2, 22.8)	22.2 (18.9, 25.3)
**Education**						
< 9th grade	4.7 (3.9, 5.5)	21.9 (18.7, 25.2)	15.3 (11.2, 18.9)	1.8 (1.1, 2.3)	2.8 (1.7, 3.8)	5.3 (3.4, 6.9)
9th–12th grade	9.3 (7.9, 10.7)	21.2 (18.6, 23.5)	13.4 (10.1, 16.7)	7.3 (5.2, 9.1)	13.2 (11.1, 15.3)	4.8 (3.2, 6.4)
HS diploma/GED	22.5 (20.7, 24.2)	24.2 (20.3, 28.0)	20.8 (17.4, 24.3)	22.1 (19.6, 24.4)	27.5 (25.5, 29.4)	13.4 (10.4, 16.2)
Some college, AD	32.3 (30.3, 34.3)	23.9 (20.6, 27.2)	28.6 (24.4, 32.9)	33.9 (29.0, 38.8)	35.4 (32.9, 37.9)	18.9 (15.6, 22.2)
≥ Bachelor's degree	31.2 (28.1, 34.3)	8.8 (6.3, 11.0)	21.9 (18.4, 25.3)	34.9 (30.0, 37.8)	21.1 (17.8, 24.3)	57.6 (52.4, 62.8)
**Poverty income ratio**						
PIR < 1.3	21.3 (19.2, 23.4)	43.1 (38.4, 47.5)	34.5 (29.2, 39.5)	15.1 (12.6, 17.3)	35.9 (31.2, 40.5)	17.2 (13.4, 20.8)
PIR 1.3 to < 3.5	36.0 (34.0, 37.9)	39.0 (34.8, 43.3)	38.9 (33.4, 44.3)	34.7 (32.2, 37.2)	40.4 (36.3, 44.4)	33.9 (28.9, 38.7)
PIR ≥ 3.5	42.7 (39.8, 45.7)	17.9 (13.9, 21.8)	26.6 (21.7, 31.5)	50.2 (46.4, 53.9)	23.8 (20.5, 27.2)	48.9 (42.6, 55.2)
**Health insurance**						
Yes	83.2 (81.5, 84.9)	56.8 (53.0, 60.4)	70.5 (66.2, 74.8)	88.4 (86.4, 90.3)	78.5 (75.6, 81.3)	87.1 (84.1, 90.0)
No	16.8 (15.1, 18.5)	43.2 (39.5, 46.9)	29.5 (25.1, 33.7)	11.6 (9.6, 13.5)	21.4 (18.6, 24.3)	12.9 (9.9, 15.8)
**Medication**						
Antihypertensive	24.8 (23.3, 26.3)	14.4 (11.6, 17.3)	18.2 (15.6, 20.9)	26.1 (23.9, 28.3)	33.1 (30.6, 25.6)	19.5 (16.8, 22.1)
Lipid-lowering	18.8 (17.5, 20.0)	12.9 (10.0, 15.0)	12.8 (10.4, 15.1)	20.4 (78.8, 22.1)	18.0 (16.2, 19.7)	18.0 (15.2, 20.9)
Antidiabetic	13.4 (12.3, 14.4)	16.5 (14.4, 19.0)	12.3 (10.8, 14.4)	12.1 (10.7, 13.5)	17.9 (18.8, 20.0)	16.5 (14.0, 19.0)

*HSD* high school diploma, *GED* general education degree, *AD* associate degree. Statistics are displayed as Percent (95% Confidence Interval).

^a^All values (except n) are weighted percentages, and age standardized to Census 2010 US population.

### Prevalence of cardiovascular disease risk factors

**Figure 2:**
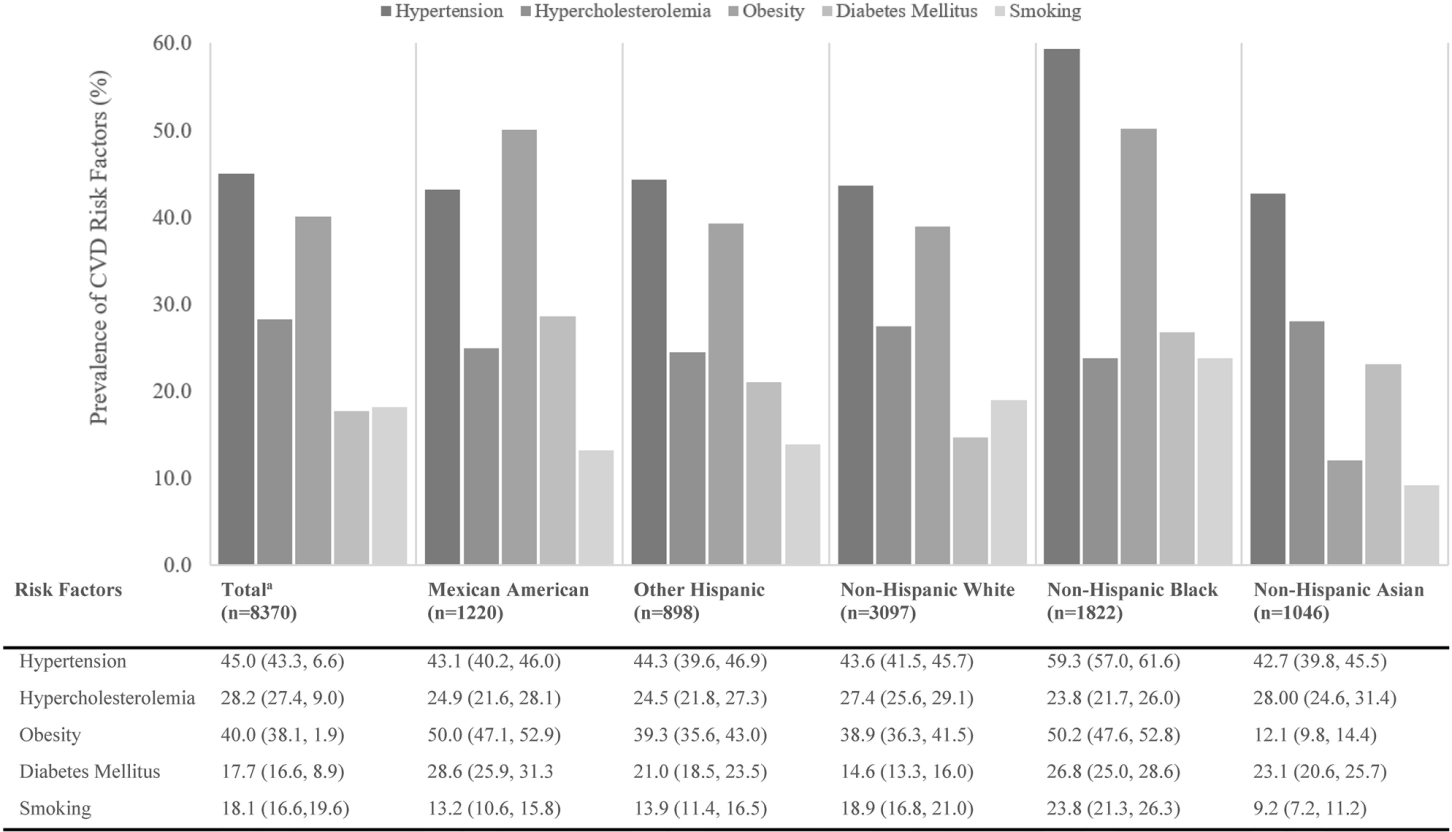
Age-standardized prevalence of cardiovascular disease risk factor for adults ≥ 20 years by race/ethnicity. *SBP* systolic blood pressure, *DBP* diastolic blood pressure, *BMI* body mass index, *FPG* fasting plasma glucose, *HbAlc* hemoglobin Alc. Risk factors defined as, hypertension (SBP ≥ 130 mmHg, DBP ≥ 80, or taking anti-hypertensive medication), hypercholesterolemia (cholesterol ≥ 240 mg/dL, or lipid-lowering medication), obesity (BMI ≥ 30 kg/m^2^), diabetes mellitus (FPG ≥ 126 mg/dL, HbAlc ≥ 6.5%, or taking hypoglycemic medication, smoking (smoked at least 100 cigarettes in life and currently smoking cigarettes). Statistics are displayed as percent (95% confidential interval). ^a^All values (except n) are weighted percentages, and age standardized to Census 2010 US population.

The prevalence of hypertension (45.0%) and obesity (40.0%) were higher in comparison to the other CVD risk factors among the participants, with three-fourths (75.0%) having at least one CVD risk factor. Hypercholesteremia was a CVD risk factor almost equally experienced by all races and ethnic groups, ranging from 24.5 to 28.0% (Fig. 2). Non-Hispanic blacks tended to have higher levels of hypertension (59.3%), obesity (50.2%), diabetes mellitus (26.8%), and be smokers (23.8%). Thus, non-Hispanic blacks were more likely than non-Hispanic Asians to have at least one CVD risk factor (83.2% and 60.9%, respectively). Half of Mexican Americans had obesity (50.0%) and a higher prevalence of diabetes mellitus compared to non-Hispanic whites (28.6% versus 14.7%, *P* < 0.001) (Fig. 2, Supplementary Table S1).

### Prevalence of cardiometabolic risk factors

**Figure 3:**
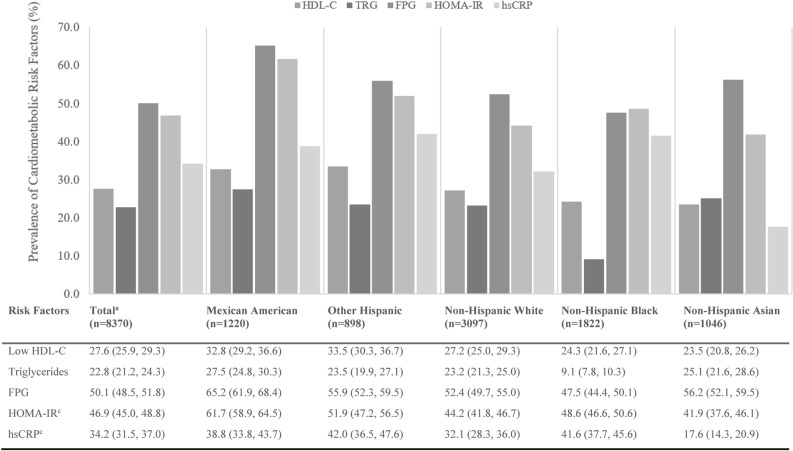
Age-standardized prevalence of cardiometabolic risk factor for adults ≥ 20 years by race/ethnicity. *FPG* fasting plasma glucose, *HDL-C* high density lipoprotein-cholesterol, *HOMA-IR* homeostatic model assessment of insulin resistance, *hsCRP* high-sensitivity C-reactive protein. Risk factors defined as, low HDL-C (< 40 mg/dL in men and < 50 mg/dL, in women), triglycerides (≥ 150 mg/dL), FPG (≥ 100 mg/dL and/or taking antidiabetic medication), HOMA-IR (≥ 2.5 [fasting glucose (mg/dL) × fasting insulin (mU/L)/405]), hsCRP (≥ 3 mg/L). Statistics are displayed as percent (95% confidential interval). ^a^All values (except n) are weighted percentages, and age standardized to Census 2010 US population

Slightly half of the participants (51.0%) had fasting plasma glucose ≥ 100 mg/dL or hypoglycemic medication use, while a little over a quarter (27.6%) had triglycerides ≥ 150 mg/dL (Fig. 3). Two-thirds (66.6%) of participants had one cardiometabolic risk factor of the three risk factors included for the summation: low HDL-C, elevated triglycerides, and glucose (Supplementary Table S3). Participants who had data for HOMA-IR (NHANES survey cycles 2011–2015) showed that the prevalence of HOMA-IR was 46.9%. Mexican Americans were more likely to have fasting plasma glucose ≥ 100 mg/dL or hypoglycemic medication use (65.2%) and triglycerides ≥ 150 mg/dL (27.5%) than the other race and ethnic groups (Fig. 3). Also, among the race and ethnic groups, Mexican Americans tended to have at least one cardiometabolic risk factor (74.0%) (Supplementary Table S3). Non-Hispanic blacks had the lowest prevalence of triglycerides (9.1%) for the race and ethnic groups.

### Prevalence of cardiovascular health metrics

The prevalence of ideal physical activity (59.2%) and smoking status (56.9%) was highest among participants while the prevalence of ideal BMI (28.5%) and diet scores (10.4%) were low (Table 3). Most participants had at least one ideal CVHM (95.2%), while 5.3% of adults had all seven ideal CVHMs (Supplementary Table S3). Of the race and ethnic groups, non-Hispanic Asians experienced more ideal CVHMs: smoking status (75.7%), BMI (56.2%), blood pressure (47.3%), and diet score (20.9%). On the contrary, non-Hispanic Asians had the lowest prevalence of ideal physical activity (47.0%) among the other race and ethnic groups. Non-Hispanic whites had a higher prevalence for ideal physical activity (62.7%), while non-Hispanic blacks had a lower prevalence for ideal diet score (8.2%). Non-Hispanic Asians were more likely to have seven CVHMs (9.9%) than Mexican Americans (4.2%) or non-Hispanic blacks (3.6%) (Supplementary Table S3).

**Table 3:** Age-standardized prevalence of cardiovascular health metrics for adults ≥ 20 years by race/ethnicity.

Metrics^a^	Total^b^ (n = 8370)	Mexican American (n = 1220)	Other Hispanic (n = 898)	Non-Hispanic White (n = 3097)	Non-Hispanic Black (n = 1822)	Non-Hispanic Asian (n = 1046)
**Smoking status**						
Ideal	56.9 (55.1, 58.7)	62.8 (59.0, 66.6)	62.4 (59.5, 65.2)	53.5 (50.9, 56.1)	59.7 (56.4, 62.8)	75.7 (72.9, 78.4)
Intermediate	25.0 (23.5, 26.6)	24.0 (20.9, 27.2)	23.7 (21.1, 26.3)	27.6 (25.4, 29.8)	16.5 (14.5, 18.6)	15.1 (12.9, 17.3)
Poor	18.1 (16.6, 19.6)	13.2 (10.6, 15.8)	13.9 (11.4, 16.5)	18.9 (16.8, 21.0)	23.8 (21.3, 26.3)	9.2 (7.2, 11.2)
**BMI**						
Ideal	28.5 (26.6, 30.3)	15.1 (12.8, 17.4)	24.4 (21.5, 27.3)	29.6 (27.2, 32.0)	21.9 (19.5, 23.2)	56.2 (52.7, 59.8)
Intermediate	31.5 (30.3, 32.8)	34.9 (32.3, 37.6)	36.3 (33.0, 39.6)	31.5 (29.6, 33.3)	27.9 (25.6, 30.2)	31.7 (28.3, 35.1)
Poor	40.0 (38.1, 41.9)	50.0 (47.1, 52.9)	39.3 (35.6, 43.0)	38.9 (36.3, 41.6)	50.2 (47.6, 52.8)	12.1 (9.8, 14.4)
**Diet score—HEI 2015**						
Ideal	10.4 (9.2 11.6)	9.7 (7.6, 11.8)	12.4 (9.8, 15.1)	10.0 (8.4, 11.7)	8.2 (6.6, 9.8)	20.9 (17.9, 23.8)
Intermediate	21.4 (19.7, 23.0)	19.8 (17.4, 22.2)	23.6 (20.2, 26.9)	21.3 (19.1, 23.4)	18.1 (16.0, 20.1)	31.1 (28.2, 34.2)
Poor	68.2 (66.2, 70.2)	70.5 (67.3, 73.8)	64.0 (59.8, 68.1)	68.7 (66.1, 71.3)	73.7 (71.0, 76.3)	48.0 (44.1, 51.8)
**Physical activity**						
Ideal	59.2 (57.7, 60.6)	52.5 (49.0, 55.9)	53.0 (47.8, 58.2)	62.7 (60.7, 64.8)	53.3 (50.8, 55.7)	47.0 (43.4, 50.6)
Intermediate	15.5 (14.2, 16.9)	12.0 (10.3, 13.8)	13.2 (11.1, 15.3)	15.5 (13.7, 17.3)	15.0 (13.1, 17.0)	21.1 (18.4, 23.8)
Poor	25.3 (23.9, 26.7)	35.5 (32.1, 38.9)	33.8 (29.3, 38.3)	21.8 (20.0, 23.6)	31.7 (28.9, 34.5)	31.9 (27.8, 36.0)
**Total cholesterol**						
Ideal	48.5 (46.8, 50.0)	49.8 (46.4, 53.2)	47.8 (44.4, 51.2)	48.0 (46.1, 49.9)	54.2 (51.9, 56.7)	46.3 (42.4, 50.2)
Intermediate	39.7 (38.1, 41.3)	39.4 (36.6, 42.2)	39.8 (36.6, 43.0)	40.0 (37.9, 42.1)	36.0 (33.8, 38.1)	41.1 (37.9, 44.4)
Poor	11.8 (10.9, 12.8)	10.8 (8.4, 13.2)	12.4 (10.0, 14.8)	12.0 (10.8, 13.3)	9.8 (8.4, 11.2)	12.6 (10.3, 14.8)
**Blood pressure**						
Ideal	41.6 (40.0, 43.4)	44.0 (41.1, 46.7)	42.1 (37.9, 46.4)	42.2 (40.1, 44.2)	30.7 (28.4, 33.1)	47.3 (44.6, 50.0)
Intermediate	24.3 (22.9, 25.7)	21.0 (18.5, 23.4)	22.0 (18.2, 25.7)	25.5 (23.7, 27.4)	22.2 (20.3, 24.1)	18.0 (15.7, 20.3)
Poor	34.1 (32.4, 35.7)	35.0 (32.5, 37.8)	35.9 (31.9, 39.9)	32.3 (30.1, 34.5)	47.1 (44.6, 49.5)	34.7 (31.8, 37.6)
**Glucose**						
Ideal	44.0 (42.0, 45.9)	32.4 (29.5, 35.2)	42.5 (38.9, 46.0)	45.5 (43.1, 48.0)	47.6 (45.2, 50.1)	41.1 (38.1, 43.9)
Intermediate	47.5 (45.6, 49.5)	54.1 (51.1, 57.1)	46.0 (42.9, 49.2)	47.2 (44.6, 49.7)	42.3 (40.0, 44.6)	49.7 (46.6, 53.0)
Poor	8.5 (7.5, 9.4)	13.5 (11.5, 15.6)	11.5 (9.7, 13.4)	7.3 (6.1, 8.5)	10.1 (8.6, 11.2)	9.2 (7.5, 10.9)
**Mean CVH score**	7.6 (7.5, 7.7)	7.3 (7.1, 7.5)	7.5 (7.2, 7.7)	7.6 (7.4, 7.7)	7.1 (7.0, 7.2)	8.8 (8.6, 8.9)
**CVH groups**						
Ideal (10-14 points)	26.9 (25.1, 28.6)	21.7 (19.2, 24.3)	26.1 (23.2, 29.0)	27.7 (25.2, 30.2)	18.6 (16.3, 20.8)	40.9 (37.6, 44.2)
Intermediate (5-9 points)	63.9 (62.3, 65.5)	65.0 (62.3, 67.6)	63.0 (59.7, 66.3)	63.8 (61.4, 66.2)	68.8 (66.1, 71.5)	56.0 (52.7, 59.1)
Poor (0-4 points)	9.2 (8.4, 10.0)	13.3 (10.6, 16.0)	10.9 (8.3, 13.5)	8.5 (7.5, 9.4)	12.6 (11.0, 14.4)	3.1 (2.0, 4.3)

*BMI* body mass index, *HEI* healthy eating index, *SBP* systolic blood pressure, *DBP* diastolic blood pressure, *CVH* cardiovascular health, *CVHM* cardiovascular health metric.

^a^Metrics defined as, smoking status: Ideal (never), Intermediate (former), Poor (current); BMI: Ideal (< 25 kg/m^2^, Intermediate (25–29.9 kg/m^2^), Poor (≥ 30 kg/m^2^); Diet-HEI 2015 score: Ideal (> 69.3), Intermediate (56.9–69.3), Poor (< 56.9); physical activity: Ideal, (≥ 150 min/wk moderate or ≥ 75 min/wk vigorous or ≥ 150 min/wk moderate + vigorous), Intermediate (1–149 min/wk moderate or 1–74 min/wk vigorous or 1–149 min/wk moderate + vigorous), Poor (none); total cholesterol: Ideal (< 200 mg/dL), Intermediate (200–239 mg/dL or treated to goal), Poor (≥ 240 mg/dL); blood pressure: Ideal (SBP < 120 and DBP < 80 mmHg), Intermediate (SBP 120–129 and DBP < 80 mmHg or treated to goal), Poor (SBP ≥ 130 or DBP ≥ 80 mmHg); Glucose: Ideal (< 100 mg/dL), Intermediate (100–125 mg/dL or treated to goal), Poor (≥ 126 mg/dL). Statistics are displayed as Percent (95% Confidence Interval).

^b^All values (except mean CVH score) are weighted percentages, and age standardized to Census 2010 US population.

### Cardiovascular health score

The mean cardiovascular health (CV) score for participants was 7.6 (95% CI 7.5, 7.7) and by race and ethnic groups ranged from 7.1 (95% CI 7.0, 7.2) (non-Hispanic blacks) to 8.8 (95% CI 8.6, 8.9) (non-Hispanic Asians). Most participants had intermediate total scores (5–9 points) (63.9%), while 26.9% had ideal total scores (10–14 points). More non-Hispanic Asians experienced ideal total scores (40.9%), while fewer non-Hispanic blacks experienced ideal total scores (18.6%, *P* < 0.001) (Table 3, Supplementary Table S2). Mexican Americans tended to categorize in the poor range group compared to non-Hispanic Asians (13.3% vs. 3.1%, *P* < 0.001, Table 3, Supplementary Table S2).

## Discussion

This study found disparities in health metrics between race and ethnic groups for hypertension, obesity, diabetes mellitus, smoking (CVD risk factors), low HDL-C, triglycerides, and fasting plasma glucose (cardiometabolic risk factors), ideal blood pressure, BMI, fasting plasma glucose, diet score, and physical activity (CVHMs). However, discrepancies did not span uniformly across the race and ethnic groups. For example, non-Hispanic blacks had a high prevalence of hypertension, obesity, diabetes mellitus, smoking status, and low prevalence of ideal blood pressure, BMI, and diet score. Yet, non-Hispanic blacks were less likely to have elevated triglycerides and more likely to experience ideal fasting plasma glucose and total cholesterol than the other race and ethnic groups. In another example, non-Hispanic whites had a much lower prevalence of obesity, diabetes mellitus, and smoking status. Non-Hispanic whites also experienced the highest prevalence of ideal physical activity, much higher even for the total participants. Mexican Americans experienced, similarly to non-Hispanic Blacks, a high prevalence of obesity, diabetes mellitus, and low prevalence of ideal diet score. Yet, Mexican Americans experienced the highest prevalence of high fasting plasma glucose, low HDL-C, and high triglycerides than the other race and ethnic groups. Finally, non-Hispanic Asians were more likely to experience at least six ideal cardiovascular health metrics in comparison to the other race and ethnic groups. This group experienced a lower prevalence of hypertension, obesity (≥ 30 kg/m^2^), diabetes mellitus, low HDL-C, and current smoking status. In addition, non-Hispanic Asians experienced a higher ideal diet score prevalence than the total sample and other race and ethnic groups. However, non-Hispanic Asians had the lowest prevalence of ideal physical activity among the race and ethnic groups.

Health insurance coverage is an important factor for individuals to receive preventive care, including health screenings, an early diagnosis and treatment, and prescription drug therapies to control chronic medical conditions, such as hypercholesterolemia^28^. Moreover, health insurance coverage often is associated with employment and income^29^. Our study showed that Mexican Americans (43.2%) and Other Hispanics (29.5%) tended to experience disproportionately higher rates of lack of insurance than the other groups, and compared to non-Hispanic whites, Mexican Americans and Other Hispanics experienced a higher poverty-to-income (PIR) < 1.3 (15.1% vs. 43.1% and 34.5%, respectively) (Table 2). Therefore, the lower health insurance coverage rates among Mexican Americans and Other Hispanics could partly explain their increased risk for CVD and cardiometabolic risk factors.

Few studies have reported the prevalence of multiple CVD risk factors in the US^4,30–32^. One study found means and proportions of CVD risk factors differences occurred over time between non-Hispanic blacks and non-Hispanic white adults participating in the NHANES^4^. In another study, African Americans participating in the National Health Interview Survey had higher self-reported CVD risk factors than African immigrants^27^. A study that included African Americans in community-based settings found that obesity and hypertension were among the common clusters of CVD risk factors^32^. Finally, CVD risk factors among US Hispanic/Latino adults participating in the Hispanic Community Health Study/Study of Latinos (HCHS/SOL) varied; 80% of men and 71% of women had at least one CVD risk factor^31^. Our study provides current prevalence estimates for the major modifiable CVD risk factors among US adults without a history of CVD and across race and ethnic groups in a nationally representative population. Additionally, our study excludes participants with CVD events: self-reported history of coronary heart failure, heart disease, angina/angina pectoris, heart attack, and stroke, strengthening the study. Our study indicates that hypertension and obesity were the most prevalent CVD risk factors among adults participating in the NHANES, with non-Hispanic blacks and Mexican Americans disproportionately experiencing those CVD risk factors, similarly reported in other studies^4,30–32^. Also, our study shows that non-Hispanic Asians had a low prevalence of smoking as a CVD risk factor, similar to findings that examined cardiovascular risk factor trends by race and ethnicity in a nationally representative adult population^4^. Our study adds to ongoing CVD surveillance efforts showing which CVD risk factors are highly prevalent among each race and ethnic group. This information is essential for knowing which race and ethnic groups need closer monitoring for CVD prevention implementation.

Few studies have reported the prevalence of cardiometabolic risk factors in a nationally representative population^7,8^. A considerable proportion of normal-weight (BMI, 18.5–24.9 kg/m^2^) adults in a nationally representative sample experienced a high prevalence of two or more cardiometabolic risk factors^8^. Also, one study showed an association between cardiometabolic risk factors and prolonged sedentary time in adults in a nationally representative population^7^. Our study provides current prevalence estimates for multiple cardiometabolic risk factors for a nationally representative population across race and ethnic groups. It shows that Mexican Americans and Other Hispanics disproportionately experienced elevated fasting plasma glucose and low HDL-C compared to non-Hispanic blacks, non-Hispanic whites, and non-Hispanic Asians. Furthermore, our study suggests that Mexican Americans and Other Hispanic with HOMA-IR and hsCRP data from the NHANES survey cycles 2011–2015 disproportionately experienced elevated HOMA-IR and hsCRP compared to the other race and other ethnic groups. Our study updates which cardiometabolic risk factors are more prevalent among each race and ethnic group and need more surveillance from a public health standpoint since these factors can lead to the development of diabetes and CVD.

Studies have reported the percentage prevalence estimates assessing cardiovascular health using the AHA-defined CVHMs among a nationally representative sample^9,10,26,33,34^. Some studies had participants stratified by age group^10,26^, by race and ethnicity differently than we stratified participants^9,26^, by gender only^33^, or by NHANES survey cycle^34^. Bundy et al.^9^ reported overall percentage prevalence estimates of CVHMs for US adults participating in NHANES 2011–2016, but participants were not free of CVD events. Whereas, our study estimates current prevalence of CVHMs for a nationally representative sample free of CVD events across race and ethnic groups using NHANES survey cycles 2011–2018. Our study shows ideal CVHMs vary for race and ethnic groups. For example, non-Hispanic whites experienced the highest prevalence for ideal physical activity, and non-Hispanic blacks experienced the lowest prevalence for ideal diet score. However, the prevalence of the ideal diet score was also low for the total sample. Also, our study uses the new guidelines for blood pressure, recognizing there are two stages for hypertension^22^, and the Healthy Eating Index-2015 (HEI-2015) score^21,35^. The HEI-2015 score is not necessarily better than the AHA score, a healthy diet score that includes five primary dietary metrics (fruits and vegetables, fish and shellfish, whole grains, sodium, and sugar-sweetened beverages)^14^. However, the HEI-2015 is an index for measuring how an individual’s dietary pattern aligns with the Dietary Guidelines for Americans and includes 13 components, food groups and nutrients of interest^21^. Thus, substituting the HEI-2015 score for the AHA score, constructed in mind for cardiovascular health^14^, is a more comprehensive approach to assessing dietary patterns.

To our knowledge, this study is the first to comprehensively examine the prevalence of estimates of CVD and cardiometabolic risk factors in conjunction with cardiovascular health for a nationally representative sample and across race and ethnic groups using the most current NHANES data. This study adds to the body of works for assessing the health of a nationally representative population across race and ethnic groups. The strengths of this study include a nationally representative US population, a sufficient sample size, the most recent estimates available to us as of now, and objective measures collected by trained personnel. A limitation of this study was the use of the Global Physical Activity Questionnaire (GPAQ) to estimate physical activity, which tends to overestimate physical activity compared to activity monitors (e.g., accelerometers and pedometers)^36^.

## Conclusion

In conclusion, US adults free of CVD events experienced hypercholesterolemia, hypertension, obesity, elevated glucose, or combinations. Moreover, few (0.8%) US adults reported seven ideal CVHMs, with few adults achieving ideal diet (10.4%) and BMI (28.5%). Our reported prevalence of CVD and cardiometabolic risk factors by race and ethnic groups provides direction for researchers, health professionals, public health, and advocacy programs to address the most urgent risk factors among these race and ethnic groups. The goal of future programs and research should be to reduce further health disparities and persisting differences among racial and ethnic groups and move toward the AHA vision for all people to have ideal cardiovascular health and live healthier longer.

## Supplementary Information

Supplementary Tables.

## Data Availability

Data used for the study are publicly available from the National Center for Health Statistics at https://wwwn.cdc.gov/nchs/nhanes/Default.aspx.
