# Supplementary material for: Racial disparities and prevalence of cardiovascular disease risk factors, cardiometabolic risk factors, and cardiovascular health metrics among US adults: NHANES 2011–2018
Source: Sci Rep. 2022 Nov 14;12:19475. doi: 10.1038/s41598-022-21878-x (PMC9663590; doi:10.1038/s41598-022-21878-x)
Supplement: Supplementary file 1 — Supplementary Tables. [file 41598_2022_21878_MOESM1_ESM.docx]

Tables S1-S3 are intended for publication as an online data supplement

|  | *P^a^* | *P* | *P* | *P* | *P* | *P* | *P* | *P* | *P* | *P* |
| --- | --- | --- | --- | --- | --- | --- | --- | --- | --- | --- |
| **Risk Factors** | **MA vs.** | **MA vs.** | **MA vs.** | **MA vs.** | **OH vs.** | **OH vs.** | **OH vs.** | **NHW vs.** | **NHW vs.** | **NHB vs.** |
|  | **OH** | **NHW** | **NHB** | **NHA** | **NHW** | **NHB** | **NHA** | **NHB** | **NHA** | **NHA** |
| **Cardiovascular Disease** | | | | | | | | | | |
| Hypertension | 0.935 | 0.748 | <.001 | 0.830 | 0.867 | <.001 | 0.796 | <.001 | 0.601 | <.001 |
| Hypercholesterolemia | 0.874 | 0.144 | 0.592 | 0.183 | 0.085 | 0.697 | 0.119 | 0.006 | 0.737 | 0.054 |
| Obesity | <.001 | <.001 | 0.815 | <.001 | 0.769 | <.001 | <.001 | <.001 | <.001 | <.001 |
| Diabetes Mellitus | 0.000 | <.001 | 0.249 | 0.002 | <.001 | 0.000 | 0.240 | <.001 | <.001 | 0.022 |
| Smoking | 0.683 | 0.001 | <.001 | 0.025 | 0.009 | <.001 | 0.005 | 0.002 | <.001 | <.001 |
| **Cardiometabolic** | | | | | | | | | | |
| Low HDL-C | 0.774 | 0.002 | 0.000 | <.001 | 0.001 | <.001 | <.001 | 0.122 | 0.026 | 0.645 |
| Triglycerides | 0.100 | 0.004 | <.001 | 0.283 | 0.879 | <.001 | 0.496 | <.001 | 0.329 | <.001 |
| FPG | 0.000 | <.001 | <.001 | 0.000 | 0.633 | <.001 | 0.892 | 0.004 | 0.070 | <.001 |
| HOMA-IR^b^ | 0.000 | <.001 | <.001 | <.001 | 0.004 | 0.196 | 0.002 | 0.004 | 0.306 | 0.002 |
| hsCRP^b^ | 0.225 | 0.500 | 0.362 | <.001 | 0.011 | 0.904 | <.001 | <.001 | <.001 | <.001 |
| **Table S1.** Age-standardized prevalence of cardiovascular and cardiometabolic risk factors for adults ≥20 years by race/ethnicity. *MA* Mexican American, *OH* Other Hispanic, *NHW* Non-Hispanic White, *NHB* Non-Hispanic Black, *NHA* Non-Hispanic Asian, *SBP* systolic blood pressure, *DBP* diastolic blood pressure, *BMI* body mass index, *FPG* fasting plasma glucose, *H1AC* hemoglobin A1c, *HDL-C* high density lipoprotein-cholesterol, *HOMA-IR* Homeostatic Model Assessment of Insulin Resistance, *hsCRP* high-sensitivity C-reactive protein, *SE* standard error, *CI* confidence interval. Risk factors defined as, hypertension (SBP ≥ 130 mmHg, DBP ≥ 80, or taking antihypertensive medication), hypercholesterolemia (total cholesterol ≥ 240 mg/dL, or lipid-lowering medication), obesity (BMI ≥ 30 kg/m^2^), diabetes mellitus (FPG ≥126 mg/dL, HbA1c ≥6.5% or taking hypoglycemic medication), smoking (smoked at least 100 cigarettes in life and currently smoking cigarettes), low HDL-C (< 40 mg/dL in men and <50 mg/dL in women), triglycerides (≥150 mg/dL), FPG (≥ 100 mg/dL and/or taking antidiabetic medication), HOMA-IR (≥2.5 [ fasting glucose (mg/dL) x fasting insulin (mU/L) /405]), hsCRP (≥ 3 mg/L). % for: categorical variables. ^a^P value calculated using Bonferroni for pairwise comparisons, accounting for multiple testing. *P* <.005 (Bonferroni, *P* <.05/by the number of comparison groups (n=10). ^b^HOMA-IR and hsCRP data available for only participants in NHANES survey cycles 2015-2016 and 2017-2018. | | | | | | | | | | |

|  | *P^b^* | *P* | *P* | *P* | *P* | *P* | *P* | *P* | *P* | *P* |
| --- | --- | --- | --- | --- | --- | --- | --- | --- | --- | --- |
| **Metrics^a^** | **MA vs.** | **MA vs.** | **MA vs.** | **MA vs.** | **OH vs.** | **OH vs.** | **OH vs.** | **NHW vs.** | **NHW vs.** | **NHB vs.** |
|  | **OH** | **NHW** | **NHB** | **NHA** | **NHW** | **NHB** | **NHA** | **NHB** | **NHA** | **NHA** |
| **Smoking Status** | | | | | | | | | | |
| Ideal | 0.962 | 0.000 | 0.172 | <.001 | <.001 | 0.157 | <.001 | 0.006 | <.001 | <.001 |
| Intermediate | 0.678 | 0.079 | <.001 | <.001 | 0.028 | <.001 | <.001 | <.001 | <.001 | 0.326 |
| Poor | 0.650 | 0.001 | <.001 | 0.034 | 0.007 | <.001 | 0.006 | 0.001 | <.001 | <.001 |
| **BMI** | | | | | | | | | | |
| Ideal | <.001 | <.001 | <.001 | <.001 | 0.004 | 0.169 | <.001 | <.001 | <.001 | <.001 |
| Intermediate | 0.568 | 0.027 | 0.000 | 0.143 | 0.019 | 0.000 | 0.066 | 0.013 | 0.876 | 0.050 |
| Poor | <.001 | <.001 | 0.815 | <.001 | 0.769 | <.001 | <.001 | <.001 | <.001 | <.001 |
| **Diet Score – HEI 2015** | | | | | | | | | | |
| Ideal | 0.107 | 0.775 | 0.307 | <.001 | 0.123 | 0.008 | <.001 | 0.103 | <.001 | <.001 |
| Intermediate | 0.078 | 0.352 | 0.282 | <.001 | 0.218 | 0.010 | 0.002 | 0.025 | <.001 | <.001 |
| Poor | 0.017 | 0.351 | 0.131 | <.001 | 0.047 | 0.000 | <.001 | 0.004 | <.001 | <.001 |
| **Physical Activity** | | | | | | | | | | |
| Ideal | 0.981 | <.001 | 0.731 | 0.030 | 0.000 | 0.836 | 0.059 | <.001 | <.001 | 0.006 |
| Intermediate | 0.507 | 0.005 | 0.008 | <.001 | 0.084 | 0.119 | <.001 | 0.863 | 0.001 | 0.002 |
| Poor | 0.740 | <.001 | 0.073 | 0.160 | <.001 | 0.281 | 0.394 | <.001 | <.001 | 0.848 |
| **Total Cholesterol** | | | | | | | | | | |
| Ideal | 0.271 | 0.204 | 0.060 | 0.128 | 0.901 | 0.003 | 0.503 | <.001 | 0.368 | <.001 |
| Intermediate | 0.601 | 0.577 | 0.083 | 0.243 | 0.950 | 0.040 | 0.514 | 0.005 | 0.457 | 0.009 |
| Poor | 0.365 | 0.332 | 0.448 | 0.293 | 0.925 | 0.079 | 0.839 | 0.005 | 0.722 | 0.051 |
| **Blood Pressure** | | | | | | | | | | |
| Ideal | 0.546 | 0.196 | <.001 | 0.071 | 0.743 | <.001 | 0.059 | <.001 | 0.003 | <.001 |
| Intermediate | 0.732 | 0.001 | 0.254 | 0.108 | 0.030 | 0.632 | 0.126 | 0.019 | <.001 | 0.008 |
| Poor | 0.735 | 0.073 | <.001 | 0.793 | 0.132 | <.001 | 0.621 | <.001 | 0.184 | <.001 |
| **Glucose** | | | | | | | | | | |
| Ideal | <.001 | <.001 | <.001 | <.001 | 0.078 | 0.004 | 0.672 | 0.180 | 0.014 | 0.000 |
| Intermediate | 0.002 | <.001 | <.000 | 0.042 | 0.835 | 0.028 | 0.120 | 0.004 | 0.152 | <.001 |
| Poor | 0.179 | <.001 | 0.002 | <.001 | 0.000 | 0.120 | 0.043 | 0.001 | 0.121 | 0.378 |
| **CVH Score** | | | | | | | | | | |
| Ideal | 0.030 | 0.002 | 0.064 | <.001 | 0.383 | <.001 | <.001 | <.001 | <.001 | <.001 |
| Intermediate | 0.376 | 0.567 | 0.040 | <.001 | 0.671 | 0.004 | 0.002 | 0.007 | 0.000 | <.001 |
| Poor | 0.224 | 0.001 | 0.686 | <.001 | 0.066 | 0.235 | <.001 | <.001 | <.001 | <.001 |
| **Table S2.** Age-standardized prevalence of cardiovascular health metrics for adults ≥20 years by race/ethnicity. *MA* indicates Mexican American, *OH* Other Hispanic, *NHW* Non-Hispanic White, *NHB* Non-Hispanic Black, *NHA* Non-Hispanic Asian, *BMI* body mass index, *HEI* healthy eating index, *SBP* systolic blood pressure, *DBP* diastolic blood pressure, *CVH* cardiovascular health, *CVHM* cardiovascular health metric, *SE* standard error, *CI* confidence interval. ^a^Metrics defined as, current smoking: Ideal (never), Intermediate (former), Poor (current); BMI: Ideal (<25 kg/m², Intermediate (25-29.9 kg/m²), Poor (≥30 kg/m^2^); Diet-HEI 2015 score: Ideal (>69.3), Intermediate (56.9-69.3), Poor (<56.9); physical activity: Ideal, (≥150 min/wk moderate or ≥75 min/wk vigorous or ≥150 min/wk moderate + vigorous), Intermediate (1-149 min/wk moderate or 1-74 min/wk vigorous or 1-149 min/wk moderate + vigorous), Poor (none); total cholesterol: Ideal (<200 mg/dL), Intermediate (200-239 mg/dL or treated to goal), Poor ((≥240 mg/dL); blood pressure: Ideal (SBP <120 and DBP <80 mmHg), Intermediate (SBP 120-129 and DBP < 80 mmHg or treated to goal), Poor (SBP ≥130 or DBP ≥80 mmHg); Glucose: Ideal (<100 mg/dL), Intermediate (100-125 mg/dL or treated to goal), Poor (≥126 mg/dL).CVH score: Ideal (10-14 pints), Intermediate (5-9 points), poor (0-4 pints). ^b^P value calculated using Bonferroni for pairwise comparisons, accounting for multiple testing. *P* <.005 (Bonferroni, *P* <.05/by the number of comparison groups (n=10). | | | | | | | | | | |

|  | **Total^a,b^**  **(n=8370)** | **Mexican American (n=1220)** | **Other Hispanic (n=898)** | **Non-Hispanic White (n=3097)** | **Non-Hispanic Black (n=1822)** | **Non-Hispanic Asian (n=1046)** |
| --- | --- | --- | --- | --- | --- | --- |
| **No. of Cardiovascular Disease Risk Factors** | | | | | | |
| 0 | 25.0 (23.4, 26.7) | 26.9 (23.5, 30.4) | 30.0 (26.5, 33.5) | 24.5 (22.3, 26.8) | 16.8 (14.8, 18.8) | 39.1 (35.6, 42.7) |
| 1 | 75.0 (73.3, 76.6) | 73.1 (69.6, 76.5) | 70.0 (66.5, 73.5) | 75.5 (73.2, 77.7) | 83.2 (81.2, 85.2) | 60.9 (57.3, 64.4) |
| 2 | 46.3 (51.8, 55.6) | 42.9 (39.2, 46.6) | 39.1 (35.2, 43.1) | 46.7 (44.2, 49.2) | 57.5 (55.0, 60.0) | 33.8 7 (30.4, 37.1) |
| 3 | 20.5 (19.3, 21.8) | 20.8 (18.0, 23.6) | 16.1 (13.1, 19.2) | 20.2 (18.6, 21.8) | 29.0 (26.4, 31.5) | 12.5 (10.4, 14.5) |
| 4 | 6.5 (5.8, 7.2) | 6.6 (5.3, 7.8) | 5.4 (3.5, 7.3) | 6.3 (5.4, 7.3) | 10.7 (9.3, 12.1) | 2.0 (1.1, 2.9) |
| **No. of Cardiometabolic Risk Factors**^c^ | | | | | | |
| 0 | 33.4 (31.5, 25.2) | 26.0 (23.2, 28.8) | 32.4 (27.8, 37.0) | 33.0 (30.7, 35.4) | 42.9 (40.4, 45.5) | 36.5 (33.6, 39.5) |
| 1 | 66.6 (64.8, 68.5) | 74.0 (71.2, 76.8) | 67.6 (63.0, 72.2) | 67.0 (64.6, 69.3) | 57.1 (54.5, 59.6) | 63.5 (60.5, 66.4) |
| 2 | 26.4 (24.5, 28.2) | 32.5 (29.0, 36.0) | 30.1 (27.0, 33.3) | 26.4 (14.0, 28.8) | 16.5 (14.5, 18.5) | 25.7 (22.8, 28.7) |
| 3 | 8.8 (7.7, 9.9) | 11.9 (9.7, 14.4) | 10.0 (7.8, 12.3) | 9.1 (7.8, 10.5) | 3.4 (2.4, 4.4) | 8.1 (6.5, 9.7) |
| **No. of Ideal Cardiovascular Health Risk Factors** | | | | | | |
| 1 | 4.8 (4.2, 5.5) | 4.3 (2.7, 5.9) | 4.2 (2.8, 5.6) | 5.3 (4.4, 5.1) | 4.5 (3.5, 5.4) | 1.7 (0.8, 2.7) |
| 2 | 95.2 (94.5, 95.8) | 95.7 (94.1, 97.3) | 95.8 (94.4, 97.2) | 94.7 (93.9, 95.6) | 95.5 (94.6, 96.5) | 98.3 (97.3, 99.2) |
| 3 | 78.6 (77.4, 79.7) | 79.0 (17.9, 24.1) | 81.7 (78.2, 85.2) | 77.3 (75.9, 78.8) | 80.0 (77.9, 82.1) | 88.3 (86.0, 90.7) |
| 4 | 55.2 (53.5, 56.8) | 57.6 (54.1, 61.1) | 60.2 (55.1, 65.3) | 53.5 (51.4, 55.6) | 54.7 (51.9, 57.5) | 69.2 (66.1, 72.2) |
| 5 | 33.4 (31.6, 35.2) | 34.7 (31.0, 38.5) | 36.5 (32.0, 41.1) | 31.9 (29.6, 34.2) | 32.0 (29.2, 34.7) | 47.9 (44.3, 51.6) |
| 6 | 16.3 (14.8, 17.8) | 16.3 (13.9, 18.7) | 17.7 (13.9, 21.4) | 16.0 (14.0, 18.0) | 13.3 (11.5, 15.1) | 27.4 (24.0, 30.8) |
| 7 | 5.3 (4.5, 6.1) | 4.2 (2.9, 5.6) | 5.7 (3.7, 7.8) | 5.3 (4.2, 6.5) | 3.6 (2.6, 4.6) | 9.9 (8.0, 11.8) |
| **Table S3.** Total number of cardiovascular disease/cardiometabolic risk factors and cardiovascular health metrics by race/ethnicity. Statistics are displayed as Percent (95% Confidence Interval). ^a^All values (except n) are weighted percentages, and age standardized to Census 2010 US population. ^b^Number of participants do not sum to 100% because the “other” race/ethnicity category (n=287) is not reported per the National Center for Health Statistics analytical guidelines. ^c^HOMA-IR and hsCRP data available for only participants in NHANES survey cycles 2015-2016 and 2017-2018. | | | | | | |
